# Comment on “Prevalence of Diabetes Mellitus and Hypertension Among COVID‐19 Patients: A Cross‐Sectional Study Exploring Associations With Sociodemographic and Biological Factors in Rajshahi Division, Bangladesh”

**DOI:** 10.1002/hsr2.71496

**Published:** 2025-11-11

**Authors:** Arooba Khan, Rafia Raza, Muddassir Khalid, Fred Segawa

**Affiliations:** ^1^ Sharif Medical and Dental College Raiwind Lahore Pakistan; ^2^ Fatima Memorial College of Medicine and Dentistry Shadman Lahore Pakistan; ^3^ Nishtar Medical University, Multan Multan Pakistan; ^4^ Makerere University College of Health Sciences Kampala Central Region Uganda

Dear Editor,

We commend the recently published article titled “Prevalence of Diabetes Mellitus and Hypertension Among COVID‐19 Patients: A Cross‐Sectional Study Exploring Associations With Sociodemographic and Biological Factors in Rajshahi Division, Bangladesh” in Health Science Reports (2025) [1]. I would like to commend the authors for their significant work, particularly relevant for low‐resource settings, which provides valuable insights into a public health issue. However, I would like to respectfully highlight several methodological concerns that could greatly affect the interpretation and relevance of the findings.

Firstly, the use of a cross‐sectional study design limits the ability to deduce significant relationships between COVID‐19 infection and the proximity of comorbidities such as diabetes mellitus and hypertension. Although correlations can be seen, some interpretations in the article are suggestive of directional effects that remain unsupported by the design employed [2].

Secondly, the sample strategy of sourcing through student networks and social media platforms carries a significant risk of selection bias. This non‐randomized sampling reduces the applicability of the results and may not accurately represent the larger group of COVID‐19 patients in the Rajshahi division or the country at large [3].

Thirdly, the classification of diabetes and hypertension was superficial. As the study categorizes participants merely as diabetic or nondiabetic, and by grouping all elevated blood pressure patients into a single category, this study neglects clinically important subgroups such as prediabetes or stages of hypertension. It may lead to loss of relevant information and also limit the accuracy of the findings [4].

Lastly, there appears to be some ambiguity in the inclusion criteria. The manuscript states that participants had diabetes mellitus, hypertension, or both, which indicates that all subjects were comorbid. However, regardless of comorbidity status, prevalence rates of these conditions are reported as if the full sample included all COVID‐19 patients. This discrepancy makes it difficult to determine the actual composition of the study population and, hence, could surely weaken the credibility of the prevalence estimates presented [5].

In conclusion, these limitations should be considered to avoid overinterpretation. Hence, future research on this topic would benefit from longitudinal designs, representative sampling approaches, more subtle variable classification, and clearer criteria for participant inclusion.

## Author Contributions

**Arooba Khan:** conceptualization, methodology, writing – original draft. **Rafia Raza:** conceptualization, methodology, writing – original draft. **Muddassir Khalid:** conceptualization, writing – review and editing, supervision, resources. **Fred Segawa:** writing – review and editing, validation.

## Conflicts of Interest

The authors declare no conflicts of interest.

## Authorship Confirmation

All authors meet the ICMJE authorship criteria and have approved the final version of the manuscript.

## Data Availability

The data that support the findings of this study are available from the corresponding author upon reasonable request.
